# Patterns and Outcomes of Complementary and Alternative Medicine Use Among Adult Patients With Multiple Sclerosis

**DOI:** 10.7759/cureus.10825

**Published:** 2020-10-06

**Authors:** Muhannad A Alnahdi, Abdullah K Alsulayhim, Ahmed H Bin Salem, Emad Masuadi, Yaser Al Malik

**Affiliations:** 1 Medicine, College of Medicine, King Saud bin Abdulaziz University for Health Sciences, Riyadh, SAU; 2 Medicine, King Abdullah International Medical Research Center, Riyadh, SAU; 3 Medical Education, College of Medicine, King Saud bin Abdulaziz University for Health Sciences, Riyadh, SAU; 4 Statistician, King Abdullah International Medical Research Center, Riyadh, SAU; 5 Neurology, King Abdulaziz Medical City, National Guard - Health Affairs, Riyadh, SAU

**Keywords:** complementary and alternative medicine, multiple sclerosis, saudi arabia

## Abstract

Multiple sclerosis (MS) is a chronic autoimmune disease that causes demyelination of the central nervous system. No treatment has been shown to be curative; thus, we assume that the tendency for patients with MS to use unconventional therapies, such as complementary and alternative medicine (CAM), might increase. The aim of this study was to explore the pattern of CAM use among patients with MS at a tertiary health care center in Saudi Arabia (SA).

This was a questionnaire-based observational cross-sectional study that targeted adult patients diagnosed with MS at King Abdulaziz Medical City in Riyadh, SA, from 2018 to 2019. The sample size included 176 patients, and a consecutive non-probability sampling technique was used to engage them during their appointments. An Arabic questionnaire was used to evaluate patients’ use of CAM.

The mean age of participants was 34.6 ± 10.9 years, females represented the majority 125 (71%) of participants, and 89% of the participants reported using CAM at least once, with one or more modalities being used. Prayer, Salat, was the most frequent modality (60%) followed by supplication, Dua'a (59%), Ruqia, reciting Holy Quran (52%), and vitamins (44%). Symptomatic improvement was reported by 49 (27.8%) of dietary supplement users and 81 (46%) of non-dietary supplement medicine users.

The study found a high prevalence of CAM utilization among Saudi adult patients with MS, which exceeded internationally reported rates. Although some patients described some improvement in their symptoms, further research is needed to evaluate the effectiveness of CAM.

## Introduction

Multiple sclerosis (MS) is an inflammatory demyelinating disease that affects the central nervous system. It is characterized by episodic disease activity disseminated in time, which produces different white matter lesions separated in space [1]. MS is considered the most common demyelinating disease, affecting more than 1one million people worldwide. North America and Europe have a high prevalence rate (>100/100,000 population), while whereas it is considerably less prevalent in the Middle East and North Africa (51.52/100,000 population) [2-3]. Patients with MS experience arrays of cognitive, motor, and sensory manifestations that can have a profound effect on their quality of life [4]. MS has no absolute cure, and although the use of immunomodulatory agents can improve the disease prognosis and prolong remission periods, their effects generally do not alleviate the symptoms or improve their quality of life [5-6].

Current MS treatment approaches can be organized into two types:, including disease-modifying therapies (DMTs) that target the underlying pathology of MS and the administration of drugs to decrease and alleviate the severity of symptoms. DMTs include drugs, such as interferon beta, sphingosine 1-phosphate (S1P) receptor modulators, such as fingolimod, and monoclonal antibodies, including natalizumab and ocrelizumab [7-10]. Patients may also be prescribed additional pharmacological interventions to alleviate their symptoms, including antidepressants or amantadine for fatigue [11-12]. While these approaches may offer some relief, MS is an incurable disease, and some patients may tend to use unconventional methods, such as complementary and alternative medicine (CAM) to help cope with their disease. There is no universal consensus on the definition and classification of CAM. The National Center for Complementary and Integrative Health (NCCIH) states that CAM practices include the use of natural products, such as herbs, vitamins, minerals, and probiotics, among others. CAM also includes the use of mind and body practices, and according to the 2012 National Health Interview Survey (NHIS), yoga, chirotherapy, and osteopathic manipulation were the most common forms of these practices [13]. In Saudi Arabia (SA), religious practices such as prayer, supplication, and cupping were reported to be part of CAM among certain Muslim populations [14].

CAM has been popular in recent years and has been employed by different populations. CAM use is widely used in Saudi Arabia (SA), though studies have varied in their estimation. A meta-analysis investigated 36 articles, which indicated the prevalence of CAM usage among the general population ranged from 21.6% to 90.5% [14]. The most frequently reported reasons for seeking CAM included failure of medical therapy, strong belief in its success, and preference of natural substances over modern medicine [14]. Several studies have indicated that up to 67% of MS patients use CAM [15-18]. In SA, CAM use among patients with MS has reportedly reached 83% [19]. Researchers also found that 43% of patients used CAM because they felt it actively contributes to their disease management, while whereas some were inspired by successful stories (22%). Notably, a relatively small minority did not consider conventional treatment to be effective (12%) [19]. These reasons might increase patients’ tendency toward the use of unconventional therapies to decrease their morbidity. The aim of this study was to explore the patterns of CAM use among patients with multiple sclerosisMS at a tertiary health care center in Riyadh, SA.

## Materials and methods

This study was conducted in specialized MS clinics at King Abdulaziz Medical City between May 2018 and June 2019 in Riyadh, SA. It was a questionnaire-based observational cross-sectional study. Study participants included patients who were >18 years old and diagnosed with clinically definite MS based on the 2010 McDonald criteria. Patients with an unclear or new diagnosis of MS were excluded from the study. Patients were consecutively approached during their appointments by the authors, and a total of 176 patients agreed to participate. Ethical approval was granted for the study from King Abdullah International Medical Research Center’s (KAIMRC’s) Institutional Review Board (IRB). The study’s objectives were explained, confidentiality and anonymity were assured, and their written consent was obtained.

The Arabic questionnaire was adapted from JaziehJeziah et al. following receipt of their approval [20]. It was further modified to match the characteristics of MSmultiple sclerosis. CAM was subdivided into dietary and non-dietary supplemental therapies. The dietary category included the use of vitamins, amino acids, and others, while whereas non-dietary modalities included religious practices, cupping, massage, chirotherapy, yoga, among others. The questionnaire considered four primary components: sociodemographic variables (e.g., age, gender, educational status), disease characteristics (e.g., disease duration, type of MSmultiple sclerosis), and the use pattern of dietary (e.g., vitamins, minerals) and non-dietary modalities (e.g., prayer, supplication).

Study variables were analyzed using the IBM Statistical Package for the Social Sciences version 24 (IBM Corp., Armonk, NY, USA) for both numerical and categorical data. Numerical variables (e.g., age) were presented as mean and standard deviation, and categorical variables (e.g., gender) were presented as frequencies and percentages. Chi-square tests were used to assess the degree of association between categorical variables (e.g., CAM usage and patients’ sociodemographic characteristics). An association with a p-value of <0.05 was considered statistically significant.

## Results

The mean age of the studied group was 34.6 ± 10.9 years, ranging from 18 to 78 years old, while and 125 participants were females. Of all participants, 71% had a university-level education, and 89% participants were married. Lack of awareness of the patients’ diagnosed MS type was found in 56% of participants. The mean disease duration was 8.1 ± 5.2 years, ranging from one 1 to 29 years. The remaining characteristics are displayed in Table 1.

**Table 1:** Sociodemographic characteristics of MS patients MS, multiple sclerosis; PP, primary progressive; RR, relapsing-remitting; SP, secondary progressive

Characteristic		N (%)
Gender	Male	51 (29%)
	Female	125 (71%)
Marital status	Unmarried	79 (47%)
	Married	89 (53%)
Education level	Secondary	49 (28.3%)
	University	124 (71.7%)
Employment status	Employed	70 (40.9%)
	Unemployed	101 (59.9%)
Patients’ MS type	RR‎/PP‎/SP	77 (43.8%)
	don't know	99 (56.3%)
Disease duration	<10 years	125 (72.3%)
	>10	48 (27.7%)

**Figure 1:**
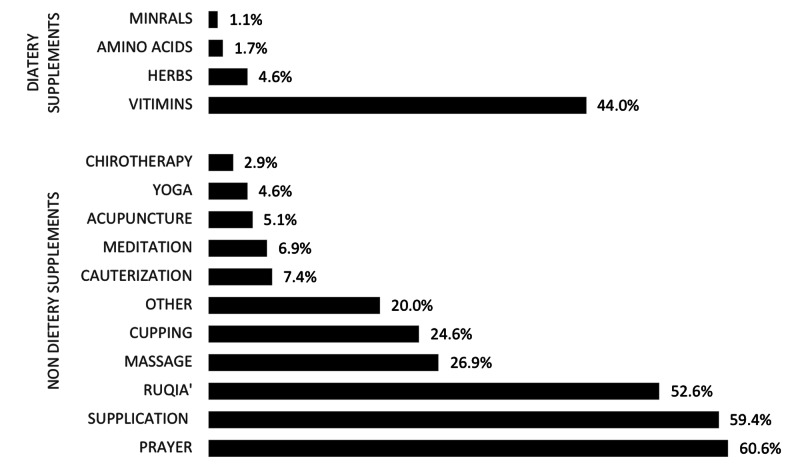
Most frequent used modalities of complementary and alternative medicine among multiple sclerosis patients (multiple selections per participant).

**Figure 2:**
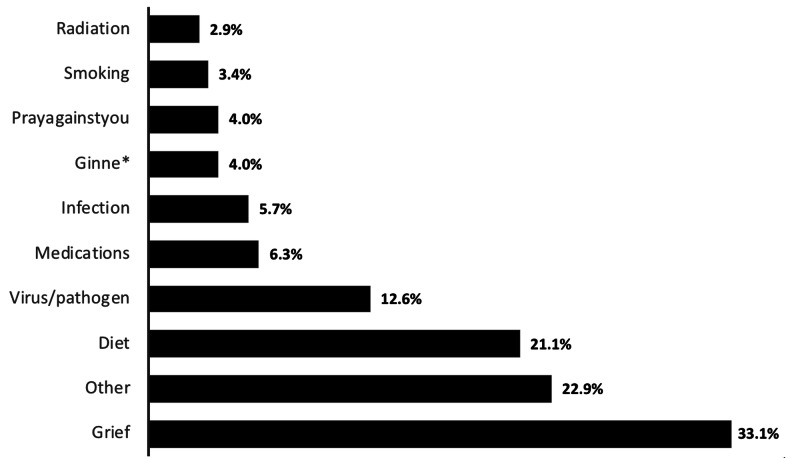
Patients’ perception of the expected causes of their multiple sclerosis illness (multiple selections per participant). *Ginne is perception of a metaphysical creature that has a superpower and can cause harm or benefit to humankind

The majority of patients (89.6%) used CAM at least once. Most frequent modalities included prayer (60.6%), supplication, Dua’a, (59.3%), Ruqia, reciting Holy Quran, (52.6%), and vitamins (44%), followed by massage (26.9%), as shown in Figure 1. Upon asking patients about their perception of the expected cause of their illness, grief was the most frequent perceived cause, followed by envy and diet, as shown in Figure 2. For symptomatic improvement, patients indicated varying degrees of perceived improvement in both dietary and non-dietary domains. Notably, 46% of patients who used non-dietary supplements indicated a perception of improvement in their psychological well-being, whereas 27.8% of patients who used dietary modalities perceived symptomatic improvement, which included a decrease in symptoms and/or improvement of overall physical well-being.

Following assessment of patients’ tendency toward disclosing their use of CAM to their treating physician, 63% of dietary supplement users indicated that had stated their disclosure, while whereas only 20% of non-dietary supplement users reported disclosure. By contrast, 33% of patients have discussed their use of non-dietary supplements with a religious counsellor or “Sheikh”. The study also revealed multiple statistically significant associations between patients who consulted a religious counselor or “Sheikh” and their characteristics, as shown in Table 2. Patients who were not aware of their particular type of MS (34.3% vs. 16.9%, p-value = 0.009), those with lower educational status (38.8% vs 22.6%; p-value = 0.031), and unemployed patients (33.7% vs. 18.6%; p-value = 0.030) were more likely to consult a non-medical counselor. Table 3 shows that the associations between CAM use and patients’ characteristics were found to be statistically insignificant. Furthermore, similar statistically insignificant results were obtained upon assessing the use of CAM subcategories and patients’ characteristics (tables are not shown).

**Table 2:** The association between status of discussing the condition with a religious counselor “Sheikh” and participants’ characteristics. ^*^Statistical significance with p-value < 0.05. MS, multiple sclerosis; PP, primary progressive; RR, relapsing-remitting; SP, secondary progressive

Variables		No	Yes	p-Value
		N (%)	N (%)	
Gender	Male	37 (72.5%)	14 (27.5%)	0.886
	Female	92 (73.6%)	33 (26.4%)	
Marital status	Unmarried	65 (82.3%)	14 (17.7%)	0.008*
	Married	57 (64%)	32 (36%)	
Educational level	Secondary	30 (61.2%)	19 (38.8%)	0.031*
	University	96 (77.4%)	28 (22.6%)	
Employment status	Employed	57 (81.4%)	13 (18.6%)	0.030*
	Unemployed	67 (66.3%)	34 (33.7%)	
Type of MS	RR‎/PP‎/SP	64 (83.1%)	13 (16.9%)	0.009*
	Don't know	65 (65.7%)	34 (34.3%)	
Disease duration	<10 years	93 (74.4%)	32 (25.6%)	0.842
	>10 years	35 (72.9%)	13 (27.1%)	

**Table 3:** The association between MS patients' sociodemographic characteristics and complementary and alternative medicine use. MS, multiple sclerosis; PP, primary progressive; RR, relapsing-remitting; SP, secondary progressive

Variables		No	Yes	p-Value
		N (%)	N (%)	
Gender	Male	5 (9.8%)	46 (90.2%)	0.893
	Female	13 (10.5%)	111 (89.5%)	
marital status	Unmarried	6 (7.7%)	72 (92.3%)	0.229
	Married	12 (13.5%)	77 (86.5%)	
Educational level	Secondary	3 (6.1%)	46 (93.9%)	0.297
	University	14 (11.4%)	109 (88.6%)	
Employment status	Employed	9 (12.9%)	61 (87.1%)	0.299
	Unemployed	8 (8%)	92 (92%)	
Type of MS	RR‎/PP‎/SP	6 (7.8%)	71 (92.2%)	0.336
	Don't know	12 (12.2%)	86 (87.8%)	
Disease duration	<10 years	15 (12.10%)	109 (87.9%)	0.261
	>10 years	3 (6.3%)	45 (93.8%)	

## Discussion

The study showed that there is an increased frequency of utilizing CAM among patients with MS in central SA. At least one modality of CAM has been used by nearly 90% of participants. This finding concurs with a previous report in eastern SA, which indicated a slightly lower percentage (83%) [19]. This percentage exceeds previous reports even on the international level, which ranged from 33 to 67% [5,15-18]. This high prevalence among the population may reflect the religious attributes that are characteristic of the Saudi community; nevertheless, other factors, such as patients’ dissatisfaction with current conventional therapies, may have resulted in patients seeking other treatment modalities [21].

The majority of participating patients were females, which is consistent with previous reports, indicating that they are more prone to MS [1,21]. Two-thirds of patients were found to have a university-level education, albeit no significant association was found with their use of CAM. Similarly, Shariff et al. reported that CAM users tended to have a university-level education [19]. These results contradict findings among American MS patients. Those patients with a postgraduate degree (e.g., doctorate degree) used CAM more frequently following their diagnosis [22]. This inconsistency strongly suggests that patients’ behaviors change across cultures and countries, along with their need to alleviate suffering.

Religious practices were the most frequently used modality. Locally, spiritual practices, such as Ruqia, prayer, reciting the Quran on water or Zamzam water, water from Zamzam well in Mecca, were previously reported to be the most commonly used modalities in the country [14]. Shariff et al. concluded that cupping and Ruqia were secondary to the use of vitamins [19]. Praying reached up to 76% among Turkish CAM users [23]. Among Germans, physical therapy and vitamins were the most used modalities, whereas Americans tended to utilize nutritional supplements, massage, special diets, and chiropractic treatment more frequently [21,24]. The religious nature of SA may have impacted the findings, leading to inconsistencies with other international studies. However, spiritual practices may seem to be a common shared point among MS patients, though it was not found to be as prevalent in SA. The American National Center for Health Statistics reported that 67.4% of MS patients used prayer for health reasons [25].

Symptomatic improvements were perceived by patients after dietary and non-dietary supplement use (27.8% and 46%, respectively). For those using dietary supplements, improvements in both symptoms and physical well-being were indicated. Although these observations were solely based on the patient assessments, a proper evaluation could help to prove these findings by objective assessment of the improvements. A previous systematic review showed that nutritional supplements might have potential benefits, which would support the stated results [26]. For non-dietary supplement users, perceived benefits were noticed by nearly half of the users. These perceived benefits tended toward better psychological and mental well-being. These findings indicated the need to further evaluate possible benefits in both CAM subcategories. Our findings were consistent with a previous report, which indicated that patients expected improvement in general health and reduction in symptoms severity, and while those were met for the most part, it was more evident in our population with non-dietary supplement users compared to dietary supplement users [27]. This may be attributable to the religious belief of the patients resulting from their spiritual affirmation that Allah holds the cure for any illness, making faith-based remedies sought after for cure, comfort, and support [20]. Therefore, randomized controlled trials are needed to confirm these results.

Patients’ knowledge of their illness type was significantly associated with their willingness to seek alternative treatment options associated with religious counselors or “Sheikh”. The lack of adequate knowledge and having an incomplete understanding of the nature of the disease are the most likely contributors to this pattern. Patients may also tend to trust healers and non-medical counselors’ promises and unproven treatments, and this may lead patients and their relatives to try other remedies. Some patients endure difficulties associated with the medication route and may experience potential adverse effects before attaining beneficial effects [28-29]. Patients indicated that CAM practitioners have specific characteristics, which were dissimilar to conventional medicine practitioners. These included listening skills, care, and concern with the patient’s empowerment [27]. This emphasizes the importance of understanding patients’ conceptions and beliefs regarding their disease. These two factors play a pivotal role in managing patients with a chronic disabling illness. These facts should direct the attention toward patients’ education regarding the nature of MS, the available treatment options, and making patients the center of care.

Following the assessment of patients’ perceptions regarding the expected causes of their illness, grief was found to be the most frequent attribute, which was followed by envy and diet. This reinforces the idea that patients may not have adequate knowledge and lack proper awareness of their disease. In eastern SA, the majority of patients had no idea about the etiology, and few mentioned lifestyle and bad habits as potential causes, whereas some even believed it was caused by an evil eye or a punishment from god [19]. One of the underestimated aspects is the necessity of tackling and uplifting patients’ psychological state and quality of life in terms of the disease perception and negative thoughts. It has been previously emphasized that depression associated with MS patients likely reaches 50%, which is higher than that experienced by patients with other chronic illnesses [30].

This study has some limitations. The sample size was relatively modest, despite the fact that the data collection was carried out for more than a year. It was also conducted in a single healthcare center in a specific region. It would be more beneficial if it was conducted on a national level by incorporating different centers located in different Saudi regions. Further studies should assess the probable contribution of culture, ethnic variability, and religious attributes, in addition to the guidelines of communicating information to patients. Furthermore, a qualitative interview-based study would complete the picture of utilizing CAM. Nevertheless, the findings of the study serve as a foundation for further studies in this field.

## Conclusions

This study evaluated the use of CAM use among patients with MS in central SA, and results showed a high prevalence of CAM utilization among patients with MS. There is a need to properly educate patients and improve their knowledge regarding CAM options, benefits, and drawbacks. Further prospective randomized controlled trials are in need to evaluate the effectiveness of complementary and alternative medicine.
